# A rare case of meconium peritonitis in a neonate: a rare clinical image

**DOI:** 10.11604/pamj.2023.45.65.39001

**Published:** 2023-05-29

**Authors:** Adithya Kiran, Yarraiahgari Maheshwara

**Affiliations:** 1 Department of Pediatrics, Jawaharlal Nehru Medical College, Acharya Vinoba Bhave Rural Hospital, Datta Meghe Institute of Medical Sciences, Sawangi, Wardha, Maharashtra, India

**Keywords:** Meconium, cystic fibrosis, Hirschsprung disease

## Image in medicine

Meconium peritonitis is an aseptic chemical inflammation caused by intrauterine bowel perforation. It is a rare but serious complication of meconium-stained amniotic fluid, occurring in 1 in 30,000 live births. It occurs when meconium enters the peritoneal cavity, usually through a defect in the bladder or a tear in the intestinal wall. Early recognition and prompt surgical intervention are crucial for a successful outcome. Perforation of the intestine in utero leads to leakage of meconium into the peritoneal cavity causing an inflammatory reaction and chemical peritonitis which subsequently seals with intra-abdominal calcifications. Possible causes include: mesenteric ischaemia, volvulus, intestinal atresia, meconium plugs, cystic fibrosis, intestinal hernias and Hirschsprung disease. Prenatal ultrasonography is diagnostic for meconium peritonitis. Diagnostic features of meconium peritonitis are abdominal calcifications, polyhydramnios, echogenic masses, meconium pseudocysts, ascites, and a dilated bowel or intestinal obstruction. We report a case of a term neonate who presented with meconium peritonitis and was successfully treated with exploratory laparotomy and antibiotics. The patient was a term neonate, male, born at 39 weeks gestational age to a primi mother via spontaneous vaginal delivery. He presented with meconium-stained amniotic fluid during delivery and was immediately intubated and suctioned. The baby was shifted to Neonatal Intensive Care Unit (NICU), started on IV fluids, and antibiotics cefotaxime and amikacin. On admission, the baby was active, tachypneic with mild intercostal retractions (Downe's score-2) with abdominal distension with a girth of 47cm and vulval edema. X-ray erect abdomen was done which showed calcifications over the liver and splenic areas with distended bowel loops (A), and ultrasound abdomen taken showed prominent large small bowel loops with internal echoes in the peritoneal cavity, suggesting bowel perforation. The diagnosis of meconium peritonitis was made. The neonate immediately underwent exploratory laparotomy with adhesiolysis with double loop ileostomy (B). The peritoneal cavity was found to contain meconium, which was suctioned out. Fecal matter with diffuse adhesions in between bowels and parietal peritoneum were also found. Dilated congested and perforated proximal bowel and collapsed distal bowel and ileal atresia at 30cm from ileo-cecal junction was noticed (C). The bladder was also drained. The neonate was closely monitored in the NICU and made a full recovery. The outcome of the disease depends on the promptness of the diagnosis and treatment, and neonates with meconium peritonitis have a high survival rate with appropriate management.

**Figure 1:**
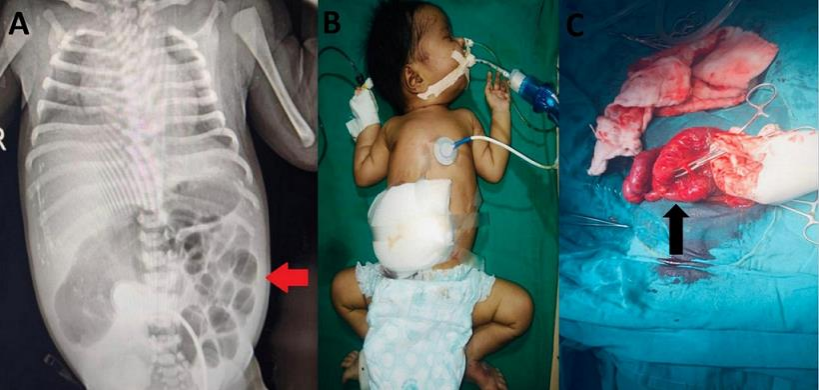
A) X-ray of the erect abdomen showing distended bowel loops and bilaterally elevated diaphragm; B) neonate with abdominal distention; C) the dilated small bowel proximal to volvulus (arrow) was resected

